# Publisher Correction: A repeating fast radio burst associated with a persistent radio source

**DOI:** 10.1038/s41586-022-05456-9

**Published:** 2022-10-31

**Authors:** C.-H. Niu, K. Aggarwal, D. Li, X. Zhang, S. Chatterjee, C.-W. Tsai, W. Yu, C. J. Law, S. Burke-Spolaor, J. M. Cordes, Y.-K. Zhang, S. K. Ocker, J.-M. Yao, P. Wang, Y. Feng, Y. Niino, C. Bochenek, M. Cruces, L. Connor, J.-A. Jiang, S. Dai, R. Luo, G.-D. Li, C.-C. Miao, J.-R. Niu, R. Anna-Thomas, J. Sydnor, D. Stern, W.-Y. Wang, M. Yuan, Y.-L. Yue, D.-J. Zhou, Z. Yan, W.-W. Zhu, B. Zhang

**Affiliations:** 1 National Astronomical Observatories, Chinese Academy of Sciences, Beijing, China; 2 Department of Physics and Astronomy, West Virginia University, Morgantown, WV USA; 3 Center for Gravitational Waves and Cosmology, West Virginia University, Morgantown, WV USA; 4 University of Chinese Academy of Sciences, Beijing, China; 5 Shanghai Astronomical Observatory, Chinese Academy of Sciences, Shanghai, China; 6 Cornell Center for Astrophysics and Planetary Science, and Department of Astronomy, Cornell University, Ithaca, NY USA; 7 Cahill Center for Astronomy and Astrophysics, California Institute of Technology, Pasadena, CA USA; 8 Owens Valley Radio Observatory, California Institute of Technology, Big Pine, CA USA; 9 Canadian Institute for Advanced Research, Toronto, Ontario Canada; 10 Xinjiang Astronomical Observatory, Chinese Academy of Sciences, Urumqi, China; 11 Research Center for Intelligent Computing Platforms, Zhejiang Laboratory, Hangzhou, China; 12 Institute of Astronomy, Graduate School of Science, The University of Tokyo, Tokyo, Japan; 13 Research Center for the Early Universe, The University of Tokyo, Tokyo, Japan; 14 Max-Planck-Institut für Radioastronomie, Bonn, Germany; 15 Kavli Institute for the Physics and Mathematics of the Universe (WPI), The University of Tokyo, Kashiwa, Japan; 16 CSIRO Space and Astronomy, Epping, New South Wales Australia; 17 School of Science, Western Sydney University, Penrith South DC, New South Wales Australia; 18 Jet Propulsion Laboratory, California Institute of Technology, Pasadena, CA USA; 19 Department of Astronomy, School of Physics, Peking University, Beijing, China; 20 Department of Physics and Astronomy, University of Nevada, Las Vegas, Las Vegas, NV USA

**Keywords:** High-energy astrophysics, Transient astrophysical phenomena

Correction to: *Nature* 10.1038/s41586-022-04755-5 Published online 8 June 2022

In the version of this article initially published, the name of author P. Wang appeared incorrectly (as P. Wan); the name has been corrected in the HTML and PDF versions of the article.

