# Supplementary material for: A repeating fast radio burst associated with a persistent radio source
Source: Nature. 2022 Jun 8;606(7916):873–7. doi: 10.1038/s41586-022-04755-5 (PMC9242862; doi:10.1038/s41586-022-04755-5)
Supplement: Supplementary file 1 — This file contains Supplementary Table 1. [file 41586_2022_4755_MOESM1_ESM.pdf]

nature portfolio

https://doi.org/10.1038/s41586-022-04755-5

Supplementary information

A repeating fast radio burst associated with a persistent radio source

In the format provided by the authors and unedited

Supplementary Information

Table 1: **Properties of the 79 FRB 20190520B bursts detected by FAST.**

Burst ID[a]	Burst time[b] (MJD)	DM[c] (pc cm^{-3})	Pulse width (ms)	Scatter tail[d] (ms)	Fluence (mJy ms)	Energy[e] ($\times 10^{37}$erg)
**P1**	58623.716958197	1238.6(20)[f]	8.7(8)	-	75(3)	9.1(4)
P2	58623.716997034	1238.6(20)	16.4(6)	-	30(2)	3.6(2)
P3	58623.717075743	1238.6(20)	7.1(8)	-	53(3)	6.4(4)
P4	58623.717421629	1238.6(20)	7.3(10)	-	51(4)	6.2(5)
**P5**	58963.766862939	1209.1(10)	10.4(2)	-	65(6)	8.0(8)
P6	58963.790991383	1209.1(10)	10.2(17)	13.1(23)	52(5)	6.4(6)
P7	58991.684351079	1214.2(5)	20.3(4)	-	107(18)	13.0(22)
P8	58991.686137870	1214.2(5)	22.8(83)	-	100(10)	12.2(12)
P9	58991.686875951	1214.2(5)	9.2(2)	-	81(12)	9.8(15)
P10	58991.704636888	1214.2(5)	19.5(17)	3.6(8)	108(12)	13.1(15)
P11	58991.704638485	1214.2(5)	9.7(7)	-	42(0)	5.2(0)
P12	58991.717697335	1214.2(5)	11.9(11)	10.3(12)	122(21)	14.9(25)
P13	58991.717878516	1214.2(5)	14.3(40)	5.1(25)	58(10)	7.1(12)
P14	58991.718217265	1214.2(5)	11.8(15)	-	63(6)	7.7(8)
P15	58991.718217670	1214.2(5)	17.5(3)	-	39(4)	4.8(5)
**P16**	58991.718218082	1214.2(5)	20.2(22)	12.6(19)	153(28)	18.6(34)
P17	58991.735450006	1214.2(5)	30.2(5)	-	138(21)	16.8(26)
P18	58991.750640038	1214.2(5)	17.7(13)	-	60(9)	7.4(11)
P19	58991.750640790	1214.2(5)	20.2(5)	-	81(14)	9.9(17)
P20	59060.484475154	1209.1(5)	13.5(13)	11.3(14)	241(12)	29.3(15)
P21	59060.507858658	1209.1(5)	7.3(9)	9.3(12)	170(8)	20.7(10)
**P22**	59060.525960600	1209.1(5)	17.3(24)	7.6(16)	167(14)	20.3(17)
P23	59061.512755579	1209.1(6)	8.5(1)	-	48(6)	5.9(7)
**P24**	59061.512755780	1209.1(6)	10.0(23)	4.7(17)	129(16)	15.7(20)
P25	59061.516277966	1209.1(6)	7.1(6)	-	29(2)	3.6(3)
P26	59061.516278904	1209.1(6)	7.5(4)	-	49(5)	6.0(7)
P27	59061.516279436	1209.1(6)	11.4(8)	-	49(4)	6.0(5)
P28	59061.524341261	1209.1(6)	10.7(8)	10.4(9)	122(16)	14.8(19)
P29	59061.535633328	1209.1(6)	14.0(4)	-	84(8)	10.2(10)
P30	59061.536568858	1209.1(6)	21.9(17)	-	72(9)	8.7(11)

Table 1: **Properties of the 79 FRB 20190520B bursts detected by FAST.**

Burst ID[a]	Burst time[b] (MJD)	DM[c] (pc cm^{-3})	Pulse width (ms)	Scatter tail[d] (ms)	Fluence (mJy ms)	Energy[e] ($\times 10^{37}$erg)
P31	59061.536569628	1209.1(6)	19.0(14)	14.0(13)	122(41)	14.8(50)
P32	59061.537903820	1209.1(6)	23.9(27)	4.9(13)	65(8)	8.0(9)
P33	59061.539298603	1209.1(6)	9.9(3)	-	73(9)	8.9(11)
P34	59061.541828100	1209.1(6)	19.8(20)	8.7(10)	212(28)	25.8(34)
P35	59067.467544345	1202.8(6)	15.2(76)	-	67(8)	8.2(10)
P36	59067.486738799	1202.8(6)	14.9(22)	17.8(29)	131(14)	15.9(17)
**P37**	59067.486739378	1202.8(6)	33.1(7)	-	146(18)	17.8(21)
P38	59067.502691880	1202.8(6)	14.3(6)	-	134(3)	16.3(3)
P39	59067.509899127	1202.8(6)	12.2(18)	10.3(17)	122(8)	14.8(9)
P40	59067.535246460	1202.8(6)	7.6(2)	-	74(9)	9.0(11)
P41	59069.495909561	1190.2(11)	16.8(5)	-	143(10)	17.4(12)
**P42**	59069.501196109	1190.2(11)	18.7(1)	-	291(14)	35.4(17)
P43	59069.514994796	1190.2(11)	12.2(15)	11.6(17)	192(13)	23.4(15)
P44	59071.472522775	1200.0(11)	5.2(3)	-	79(1)	9.6(2)
P45	59071.472523007	1200.0(11)	7.2(7)	-	66(1)	8.1(2)
**P46**	59071.491696655	1200.0(11)	10.4(20)	4.9(15)	184(4)	22.4(5)
P47	59073.496887082	1197.0(7)	7.8(2)	-	107(9)	13.1(11)
**P48**	59073.515256071	1197.0(7)	18.7(6)	-	147(2)	17.9(2)
P49	59073.515256881	1197.0(7)	11.1(7)	-	57(4)	7.0(5)
**P50**	59075.454353002	1210.6(6)	24.9(1)	-	313(19)	38.0(23)
P51	59075.454862469	1210.6(6)	22.0(1)	-	209(12)	25.5(15)
P52	59075.472181012	1210.6(6)	14.0(5)	-	114(6)	13.8(8)
P53	59075.484186304	1210.6(6)	6.0(0)	-	157(16)	19.1(20)
P54	59075.496463775	1210.6(6)	16.7(4)	-	83(8)	10.1(9)
P55	59077.448938437	1209.9(4)	11.9(5)	-	103(7)	12.5(8)
P56	59077.448939131	1209.9(4)	14.4(7)	-	107(5)	13.1(7)
P57	59077.449538432	1209.9(4)	13.4(17)	10.6(16)	199(10)	24.2(12)
P58	59077.449939184	1209.9(4)	5.4(26)	-	148(7)	18.0(8)
P59	59077.449939372	1209.9(4)	9.6(29)	-	121(11)	14.7(14)
P60	59077.460100338	1209.9(4)	13.0(23)	13.5(28)	132(1)	16.1(2)
P61	59077.460503968	1209.9(4)	10.3(18)	10.6(21)	94(5)	11.4(6)
P62	59077.466295203	1209.9(4)	11.7(2)	-	156(10)	19.0(12)

Table 1: **Properties of the 79 FRB 20190520B bursts detected by FAST.**

Burst ID[a]	Burst time[b] (MJD)	DM[c] (pc cm^{-3})	Pulse width (ms)	Scatter tail[d] (ms)	Fluence (mJy ms)	Energy[e] ($\times 10^{37}$erg)
P63	59077.468715062	1209.9(4)	18.4(35)	7.6(18)	128(9)	15.6(11)
P64	59077.469903193	1209.9(4)	11.2(10)	11.3(12)	152(7)	18.5(9)
P65	59077.475047947	1209.9(4)	11.0(17)	8.3(16)	107(6)	13.0(8)
P66	59077.475330491	1209.9(4)	14.0(8)	12.9(9)	205(11)	24.9(14)
P67	59077.477447949	1209.9(4)	2.0(1)	-	70(2)	8.5(3)
**P68**	59077.485451841	1209.9(4)	14.3(1)	-	332(7)	40.4(8)
P69	59077.485451957	1209.9(4)	14.4(1)	-	262(15)	31.8(19)
P70	59077.490027004	1209.9(4)	8.4(4)	-	132(2)	16.1(3)
P71	59077.491413589	1209.9(4)	10.3(3)	-	115(5)	14.0(6)
P72	59077.497806611	1209.9(4)	15.3(22)	10.7(21)	138(9)	16.8(11)
P73	59077.497957805	1209.9(4)	13.6(4)	-	89(5)	10.9(6)
P74	59077.498960966	1209.9(4)	4.6(7)	11.8(15)	94(6)	11.4(7)
**P75**	59089.428163703	1186.7(25)	11.2(2)	-	112(3)	13.6(4)
P76	59089.436165435	1186.7(25)	13.3(6)	-	83(2)	10.1(3)
**P77**	59111.370525959	1183.3(17)	20.6(6)	-	99(11)	12.0(14)
P78	59111.370526098	1183.3(17)	6.7(1)	-	106(8)	12.9(9)
P79	59111.370978062	1183.3(17)	14.6(5)	-	98(9)	11.9(11)

[a] The burst IDs are from 1 to 79. P1 to P4 were detected in the drift scan mode. P4 to P79 were detected in the tracking mode.

[b] Arrival time of burst at the solar system barycenter in barycentric dynamical time (TDB). They are corrected to the frequency of 1.5 GHz in the International Celestial Reference System (ICRS).

[c] All the bursts detected on the same day are assigned the best fit DM value of the highest S/N burst from that day; the highest S/N burst ID is shown in bold for each observing epoch. Epochs are separated by single horizontal lines and the apparent DM variations may solely be the result of the variable spectra-temporal structure of the bursts.

[d] We only report burst scattering timescales with fractional uncertainties less than 50%. These scattering times are based on fitting a Gaussian pulse convolved with a one-sided exponential to the 1D burst profile, which was averaged over the entire burst bandwidth.

[e] Energy here refers to equivalent isotropic energy.

[f] Reported uncertainties correspond to the last consecutive digits; e.g., $1238.6(20) = 1238.6 \pm 2.0$.
